# Prognostic value of dysadherin in cancer: A systematic review and meta-analysis

**DOI:** 10.3389/fonc.2022.945992

**Published:** 2022-09-02

**Authors:** Aino Niinivirta, Tuula Salo, Pirjo Åström, Krista Juurikka, Maija Risteli

**Affiliations:** ^1^ Cancer and Translational Medicine Research Unit, Faculty of Medicine, University of Oulu, Oulu, Finland; ^2^ Medical Research Center Oulu, Oulu University Hospital, University of Oulu, Oulu, Finland; ^3^ Department of Oral and Maxillofacial Diseases, University of Helsinki, and Helsinki University Central Hospital, Helsinki, Finland; ^4^ Department of Pathology (HUSLAB), Helsinki University Central Hospital, University of Helsinki, Helsinki, Finland; ^5^ Translational Immunology Research Program (TRIMM), University of Helsinki, Helsinki, Finland; ^6^ Research Unit of Biomedicine, Faculty of Medicine, University of Oulu, Oulu, Finland

**Keywords:** cancer, dysadherin, prognosis, systematic review, meta-analysis

## Abstract

Cancer is a leading cause of death worldwide and novel prognostic factors are reported with increasing numbers. Systematic reviews and meta-analyses on cumulative research data are crucial in estimating the true prognostic value of proposed factors. Dysadherin (FXYD Domain Containing Ion Transport Regulator 5; FXYD5) is a cell membrane glycoprotein that modulates Na+, K+-ATPase activity and cell-cell adhesion. It is abundantly expressed in a variety of cancer cells, but only in a limited number of normal cells and its levels are increased in many different tumor types. The expression or level of dysadherin has been suggested as an independent predictor for metastasis and poor prognosis by number of studies, yet we lack a definitive answer. In this study, we systematically evaluated the prognostic value of dysadherin in cancer and summarized the current knowledge on the subject. PubMed, Scopus, Web of Science and relevant clinical trial and preprint databases were searched for relevant publications and PRISMA and REMARK guidelines were applied in the process. After a careful review, a total of 23 original research articles were included. In each study, dysadherin was pointed as a marker for poor prognosis. Meta-analyses revealed 3- and 1.5-fold increases in the risk of death (fixed effects HR 3.08, 95% CI 1.88-5.06, RR 1.47, 95% CI 1.06-2.05 on overall survival, respectively) for patients with high (>50%) tumoral FXYD5 level. In many studies, a connection between dysadherin expression or level and metastatic behavior of the cancer as well as inverse correlation with E-cadherin level were reported. Thus, we conclude that dysadherin might be a useful prognostic biomarker in the assessment of disease survival of patients with solid tumors.

## Introduction

Dysadherin, also known as FXYD domain-containing ion transport regulator 5 (FXYD5), DYSAD, or IWU1, is a member of FXYD family, of which all seven members (FXYD1–7) are known to interact with Na+/K+-ATPase and tissue-specifically modulate its function. Dysadherin is a cell membrane glycoprotein abundant only in a limited number of normal cells - namely lymphocytes, endothelial cells, and cells of epithelial tissues (1). FXYD5 gene locates in chromosome 19 and encodes a 178 amino acid protein with a putative signal sequence, a potential O-glycosylated extracellular domain, a single transmembrane domain, and a short cytoplastic tail (2) (3). To our knowledge, there are no human disease conditions or animal models of disease deriving from mutations in FXYD5 (4). However, for many different cancer types, increased dysadherin expression is an independent predictor of metastasis and poor prognosis (1). The use of FXYD5 expression as a prognostic factor has not been studied in other pathologies than cancer. In general, dysadherin is not yet an extensively studied molecule, and its clinical usability and full biological function are just being discovered.

Initially dysadherin was identified as a target of a monoclonal antibody which was developed to react with a variety of cancer cells, but only with few normal cells (3). In normal cells, dysadherin has a physiological role in modulating cellular junctions, affecting cell adhesion, influencing chemokine production and, most importantly, modulating Na+/K+-ATPase activity (1). In addition, dysadherin has a role in normal epithelia during inflammation. Lubarski-Gotliv et al. demonstrated that in epithelium, dysadherin increases the cells’ response to lipopolysaccharide (LPS) through tumor necrosis factor α (TNF-α) signaling (5). Brazee et al. studied the pro-inflammatory effects of dysadherin in lung injury and found dysadherin to be one of the key contributors in the pulmonary inflammatory response (6). Recently, dysadherin was shown to be a substrate of MMP8 and its cleavage increased cell-cell adhesion leading to restrained migration in oral cancer cells (7).

Dysadherin is linked to certain known cancer promoting signaling pathways. High expression of dysadherin is involved in the downregulation of E-cadherin, which by acting as the cell-cell adhesion receptor, has an important role in suppression of tumor progression (1). Downregulation of E-cadherin leads to reduced cell adhesion and upregulation of chemokine production thereby creating more favorable conditions for metastatic spread (8). However, dysadherin expression also correlates with changes in cell morphology and increased metastasis in cancer cells lacking E-cadherin expression, which suggests that dysadherin also exerts E-cadherin independent mechanisms in tumor progression (1). Moreover, dysadherin expression increases the secretion of chemokine (C-C motif) ligand 2 (CCL2) by enhancing the transcriptional activity of NF-κB (9) and increasing the activation of AKT (10) *in vitro*.

The previous review articles on the role of dysadherin in cancer prognosis are not systematic reviews on the subject and are not considering solely cancer prognosis (1, 8, 11, 12). The review article by Lubarski et al. gathered the current knowledge on FXYD5 mostly focusing on its functional effects and experimental data (1). A review by Nam et al. focused on the possible mechanisms of FXYD5 in the process of cancer progression (8). They concluded that dysadherin is a potential molecular target for the visualization, prevention or treatment of head and neck cancers with advanced stage. Molecular mechanisms of FXYD5, mostly related to E-cadherin regulation was discussed in the review by Georgolios et al. (12). Articles examining dysadherin in head and neck cancers specifically were reviewed by Giotakis et al. (11). They concluded that dysadherin is frequently overexpressed in head and neck cancer and could be a potential, reliable independent prognostic factor. By applying systematic review guidelines and methods, we gathered the original research articles on dysadherin related to cancer prognosis and provided a comprehensive compilation of the current knowledge on the subject. Thus, we aimed to create an overview of the prognostic value of dysadherin in cancer.

## Materials and methods

### Search protocol

This article was compiled by following the systematic review guide – Preferred Reporting Items for Systematic Reviews and Meta-Analyses (PRISMA 2020) (13). Searches were performed in PubMed (https://pubmed.ncbi.nlm.nih.gov), Scopus (https://www.scopus.com), Web of Science (https://www.webofscience.com), clinical trial databases clinicaltrials.gov (https://clinicaltrials.gov/) and ICTRP (https://www.who.int/clinical-trials-registry-platform) as well as pre-print databases MedRXiv/BioRXiv (https://www.medrxiv.org/) and ResearchSquare (https://www.researchsquare.com/). All reports published until July 2022 were included. The search terms dysadherin (dysadherin OR dysad OR fxyd5 OR iwu1) and cancer (cancer OR tumo?r* OR neoplasm* OR carcinoma OR malignan* OR sarcoma* OR leukemi* OR lymphoma OR adenocarcinoma*) and prognosis (prognos*) were searched from titles, abstracts and keywords (Scopus) or all fields (PubMed, Web of Science). Clinical trial databases were searched for dysadherin (as above) and preprint databases with dysadherin (as above) as well as prognosis (prognos*) due to limitations in search query length. The asterisk is used to indicate truncation and the question mark to indicate wildcard characters in search terms.

### Review of relevant publications

**Figure 1:**
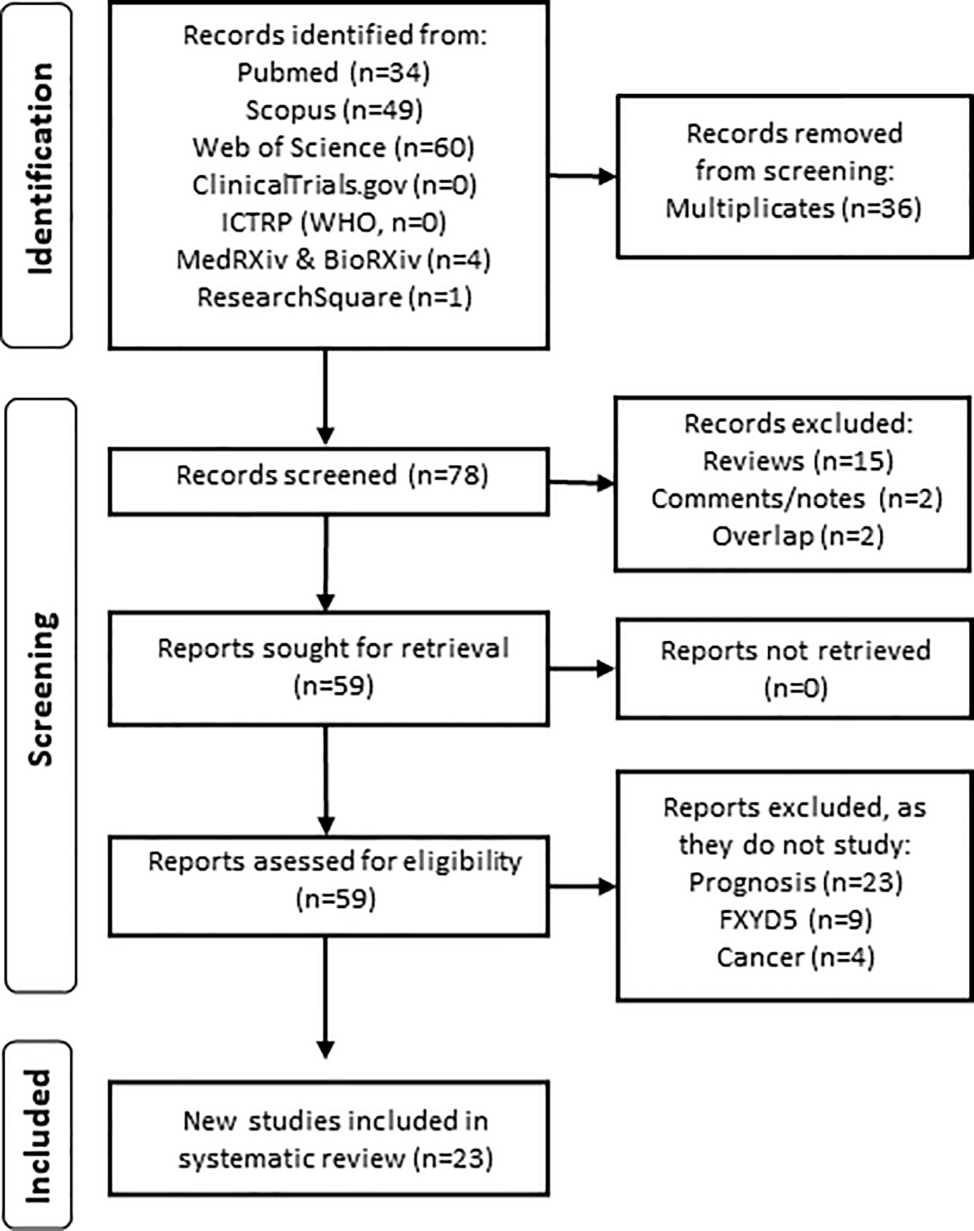
Systematic literature search depicted as flowchart.

The workflow of the systematic review is depicted in **Figure 1**. A total of 148 search hits were retrieved originally. After removing the multiplicate articles, 78 articles remained, of which articles other than original research (reviews or comments/notes) or overlapping (same report in conference and original publication) were excluded. The remaining 59 reports were carefully retrieved and analyzed. Reports were further excluded if dysadherin or cancer was not studied, or if the study did not focus on prognostic value of dysadherin in cancer. Two reviewers (AN and KJ) independently screened and assessed the selected literature. Differences in the results, if any, were resolved through discussion with a third reviewer (MR). Finally, 23 collected reports were included in this systematic review. To answer our specific research question (“What is the prognostic value of dysadherin in cancer?”) the information about study size and type, main findings and statistical methods were retrieved. All extracted data is presented shortly in the **Table 1** (reference, cancer type, effect on prognosis and statistics) and fully in the **Supplementary Table 1** (reference, country where and years when study was conducted, received therapies, sample type, antibody used, correlation with patient parameters and period of follow up in months).

**Table 1:** Overview of reports (included in the systematic review) studying dysadherin in evaluating cancer prognosis.

First author, year	Cancer type	Total sample number (for prognosis)	Effect on prognosis (survival) and statistical method
Aoki et al. (2003) (14)	Colorectal carcinoma	82 IHC	High protein level predicts poor overall (multivariate HR 3.50, 95% CI 1.03-11.85, p 0.044) and recurrence-free (multivariate HR 2.52, 95% CI 1.06-5.98, p 0.036) survival. Kaplan-Meier, log-rank, Cox regression.
Jin et al. (2021) (15)	Colon cancer	455 TCGA	High mRNA expression predicts poor overall (univariate HR 1.76, 95% CI 1.18-2.62) and progression-free (univariate HR 1.69, 95% CI 1.17-2.44) survival. Kaplan-Meier, log-rank, Cox regression.
Park et al. (2022) (16)	Colorectal carcinoma	105 IHC	High protein level predicts poor overall (multivariate HR 3.86, 95% CI 1.70-8.79) and recurrence-free (multivariate HR 2.57, 95% CI 1.05-6.31) survival in stage II-III patients. Kaplan-Meier, log-rank, Cox regression.
Shimamura et al. (2003) (17)	Pancreatic ductal adenocarcinoma	125 IHC	High protein level predicts poor overall survival (multivariate HR 2.17, 95% CI 1.14-4.14, p 0.019, <20% vs >51%). Kaplan-Meier, log-rank, Cox regression.
Shimada et al. (2004a) (18)	Gastric cancer	276 IHC	High protein level predicts poor overall survival (univariate, log-rank p 0.002, Wilcoxon p 0.001). Kaplan-Meier, log-rank, Wilcoxon, Cox regression.
Wu Z et al. (2020) (19)	Extrahepatic cholangiocarcinoma	155 IHC	High protein level predicts poor overall survival (univariate HR 3.26, 95% CI 2.079-5.102, p 0.000; multivariate HR 2.09, 95% CI 1.25-3.51, p 0.005). Kaplan-Meier, log-rank, Cox regression.
Shimada et al. (2004b) (20)	Esophageal squamous cell carcinoma	117 IHC	High protein level predicts poor overall survival (univariate p 0.003, multivariate RR 2.57, 95% CI 1.40-4.71, p 0.003). Kaplan-Meier, log-rank, Cox regression.
Tian et al. (2021) (21)	Renal cancer	525 TCGA, GEO	As part of a seven gene set, high mRNA expression of which predicts poor overall survival (univariate HR 4.27, 95% CI 3.09-5.91, p<0.001; multivariate HR 3.78, 95% CI 2.56-5.59, p<0.001). Cox regression.
Raman et al. (2015) (22)	Ovarian cancer	572 TCGA	High mRNA expression predicts poor overall survival (univariate log-rank p 0.000, multivariate HR 1.16, p 0.020). Kaplan-Meier, log-rank, Cox regression.
Tassi et al. (2019) (23)	Serous ovarian cancer	68 mRNA, 39 microarray, 48 IHC, 1341 TCGA, curatedOvarianData	High mRNA expression (univariate HR 2.09, 95% CI 1.19–3.69, p 0.011; multivariate 1.93, 95% CI 1.08–3.45, p 0.025) and protein level (univariate HR 2.57, 95% CI 1.24–5.32, p 0.011; multivariate 2.30, 95% CI 1.10–4.80, p 0.026) predicts poor overall survival. High mRNA expression (univariate HR 1.97, 95% CI 1.16-3.33, p 0.012; multivariate HR 1.92, 95% CI 1.13-3.25, p 0.016) and high protein level (univariate HR 2.18, 95% CI 1.15-4.14, p 0.017; multivariate HR 2.11, 95% CI 1.11-4.02, p 0.023) predicts poor progression-free survival. Kaplan-Meier, Cox regression.
Bai et al. (2020) (24)	Ovarian cancer	58 IHC, 655 TCGA	High mRNA expression predicts poor overall (univariate HR 1.59, 95% CI 1.26-2.00, p 0.000), relapse-free (univariate HR 1.69, 95% CI 1.37-2.08, p 0.000) and post-progression (univariate HR 1.58, 95% CI 1.24-2.01, p 0.00018) survival. Kaplan-Meier, log-rank.
Wu et al. (2004) (25)	Cervical squamous cell carcinoma	206 IHC, 20 mRNA	High protein level predicts poor overall survival (univariate log-rank p 0.04). Kaplan-Meier, log-rank, Cox regression.
Besso et al. (2019) (26)	Endometrial cancer	32 mRNA, 332 TCGA	As part of a four gene panel, high mRNA expression predicts poor overall survival (univariate HR 2.05, 95% CI 1.05-4.17, p 0.048). Kaplan-Meier, log-rank.
Nakanishi et al. (2004) (27)	Tongue cancer	91 IHC	High protein level predicts poor overall survival (multivariate HR 2.68, 95% CI 1.40-15.13, p 0.003). Kaplan-Meier, log-rank, Cox regression.
Kyzas et al. (2006) (28)	Head and neck squamous cell carcinoma	108 IHC	High protein level predicts poor overall survival (univariate HR 4.84, 95% CI 1.95–11.99, p<0.001; multivariate 3.92, 95% CI 1.46–10.51 p 0.006). Kaplan-Meier, log-rank, Cox regression.
Muramatsu et al. (2008) (29)	Head and neck cancer	48 IHC	(No significant correlation to DFS). Kaplan-Meier, generalized Wilcoxon.
Chen et al. (2021) (30)	Head and neck squamous cell carcinoma	256 TCGA, GEO	High mRNA expression predicts poor overall survival (univariate p 0.015). Kaplan-Meier, Cox regression. *
Sato et al. (2003) (31)	Thyroid carcinoma	92 IHC	High protein level in patients who died of thyroid carcinoma (p<0.001). Mann-Whitney U.
Tamura et al. (2005) (32)	Non-small cell lung cancer	131 IHC	High protein level predicts poor overall survival (univariate p 0.006; multivariate HR 3.02, 95% CI 1.75-2.01, p 0.010). Kaplan-Meier, log-rank, Cox regression.
Ono et al. (2010) (33)	Non-small cell lung cancer	107 IHC	High protein level predicts worse disease-free survival (univariate HR 2.62, 95% CI 1.08-6.33, p 0.032; multivariate HR 2.95, 95% CI 1.10-7.94, p 0.032). High protein level together with low E-cadherin level predicts poor overall (univariate p 0.012) and progression-free (univariate p 0.039) survival. Kaplan-Meier, logrank, Cox regression*.
Nishizawa et al. (2005) (34)	Melanoma	115 IHC	High protein level predicts poor overall survival (multivariate HR 18.98/17.58, 95% CI 4.02-89.51/3.99-77.45, both p<0.001, score + and 2+ respectively). Kaplan-Meier, log-rank, Cox regression.
Izumi et al. (2006) (35)	Epithelioid sarcoma and malignant rhabdoid tumor	78 IHC	High protein level predicts poor overall survival in epithelioid sarcoma (univariate p 0.000; multivariate p 0.000). Kaplan-Meier, log-rank, Cox regression.
Izumi et al. (2007) (36)	Synovial sarcoma	92 IHC	High protein level predicts poor overall survival (univariate p 0.001; multivariate p 0.041). Kaplan-Meier, log-rank, Cox regression.

IHC, Immunohistochemistry; TCGA, The Cancer Genome Atlas; GEO, Gene Expression Omnibus; HR, Hazard ratio; RR, Risk ratio; CI, Confidence interval. *Please note that there is a discrepancy between text and images or in the text details

### Evaluation of publication quality

We used the Reporting Recommendations for Tumor Marker Prognostic Studies (REMARK) guidelines to evaluate the quality of reporting in each study (37). The REMARK evaluations are presented in the **Supplementary Table 2** and evaluation of inter-rater agreement with Cohen’s κ showed substantial agreement (0.72) between reviewers. The REMARK criteria consist of a checklist with 20 items of which we eliminated three irrelevant items for this kind of studies, leaving us with a checklist of 16 items. The eliminated items were 6, 8, 9, and 18. The item was fulfilled if the article reported most of the criteria listed. Items 4 and 13, describing the control samples, and reporting the marker expression against standard prognostic variables respectively, were missing from several articles. Discussion about limitations of the study (in item 19) was missing from almost all articles.

### Meta-analysis of relevant publications

The open software R (version 4.1.2) with the package “metafor” (version 3.0-2) was used for meta-analysis. By utilizing the rma.uni-function (i.e. inverse variance method), both fixed and random effects models were applied to estimate the combined effect of FXYD5 expression on overall survival in selected studies. The heterogeneity statistics Cochran’s Q and I2 are presented to describe the variance between included studies. The p-value < 0.05 is considered statistically significant.

## Results

As shown in **Figure 1**, our original search resulted in 148 hits in seven databases. After removing the multiplicates (n=36) and reports without original data (n=19), we were left with 59 reports, which were retrieved and analyzed. Finally, 23 reports which provided data on the connection of dysadherin expression/level and cancer patients’ survival were deemed eligible. **Table 1** contains an overview of the reports included in this study. In general, we only discuss the statistically significant results identified from the included studies and exact statistical values are reported in **Table 1**. In **Supplementary Table 1** additional clinical details of the studies are listed.

For this systematic review, we classified articles according to the anatomical location of the tumor into five categories: gastrointestinal and urinary, gynecological, head and neck, lung, and skin and connective tissue cancers. In addition, results from meta-analyses on the use of dysadherin protein level as prognostic marker are included as a separate chapter.

### Gastrointestinal and urinary cancers

The tumoral dysadherin has been evaluated in gastrointestinal and urinary cancers in eight studies reporting high dysadherin protein level and mRNA expression as a marker of poor overall survival (14–21) (**Table 1** and **Supplementary Table 1**). Dysadherin protein was immunohistochemically observed on cancer cell membranes, but not in the normal epithelium in colorectal carcinoma (n=82 and n=123, 2 studies), gastric cancer (n=276), pancreatic ductal adenocarcinoma (PDAC, n=125) and extrahepatic cholangiocarcinoma (100 ECC, 30 peritumoral tissues, 10 adenoma, 15 normal biliary tract tissues) (14, 15, 17–19). In contrast, in esophageal squamous cell carcinoma (ESCC, n=117) dysadherin staining was also observed in the basal cells of normal epithelium (20). Similarly, Tian et al. identified high FXYD5 mRNA expression to predict poor overall survival in renal kidney cancer as part of a seven-gene signature (21). An association between dysadherin protein level and metastasis was found in colorectal carcinoma, ECC, PDAC and gastric cancer (14, 17–19).

### Gynecologic cancers

The role of dysadherin in gynecologic cancers has been investigated in five studies, all reporting high dysadherin mRNA expression or protein level as a marker of poor overall survival (22–26) (**Table 1** and **Supplementary Table I**). Raman et al. examined potential survival-related markers by utilizing copy number amplifications and gene expression datasets of serous ovarian carcinomas (SOC) (n=572) in The Cancer Genome Atlas (TCGA). Dysadherin was identified as a marker of poor survival in SOC, and this finding was validated in another set of microarray data from ovarian carcinomas (OC) (n=204) (22). Tassi et al. compared the survival biomarkers between long-term and short-term high-grade SOC survivors (n=39 in training set, n=29 in validation set). Dysadherin was upregulated both on mRNA and protein level in patients with poor overall survival compared to those showing favorable outcome. Multivariate analysis revealed dysadherin as an independent prognostic factor of mortality (23). Bai et al. studied patients with high-grade stage III OC (n=58) and patients with benign ovarian tumors or uterine lesions (n=22). Using immunohistochemistry, the authors demonstrated higher dysadherin level in OC patients’ tumors compared to normal tissues. Upregulation of dysadherin mRNA associated with a poor overall, relapse-free and post-progression survival in epithelial ovarian cancer patients analyzed with Kaplan-Meier Plotter tool (24).

In cervical squamous cell carcinomas (CSCC, n=206), higher protein level of dysadherin was significantly associated with shorter overall survival. Wu et al. found that most tumors were positive for dysadherin protein, and that dysadherin was also present in the basal and parabasal cells of normal cervical epithelia (25). In addition, Besso et al. evaluated dysadherin in tumor samples (n=74) and concluded that high mRNA expression in tumors were associated with endometrial cancer aggressiveness. Analysis of uterine corpus endometrioid cancer patients (TCGA) identified a high-risk group of patients with increased dysadherin mRNA expression and shorter overall survival rates compared to the low-risk group (26).

### Head, neck and thyroid cancers

Five studies examined dysadherin in head and neck cancers (27–31), and three of them suggested high tumoral dysadherin mRNA expression or protein levels to predict poor overall survival (**Table 1** and **Supplementary Table 1**). Muramatsu et al. evaluated the impact of dysadherin on survival of head and neck cancer patients (n=48) treated with radiation therapy. Patients with high dysadherin levels showed a poor response to radiation therapy, but no correlation to disease free survival or the recurrence were found. Dysadherin protein level alone did not correlate with metastasis but correlation to metastasis was found when it was combined with E-cadherin staining level (29). Chen et al. identified dysadherin as one of the immune-related differentially expressed genes between human papilloma virus positive (HPV+) and negative (HPV-) head and neck cancer patients. High dysadherin mRNA expression was included in a nine immune-gene panel, which could separate HNSCC patients in to high- and low risk groups with poor and better prognosis, accordingly (30).

Nakanishi et al. showed association of high dysadherin level with tumor stage and infiltrative growth pattern in tongue squamous cell carcinoma (n=91). Dysadherin-positive staining was observed on the membranes of cancer cells as well as in the basal cells of normal squamous epithelium. Increased dysadherin immunopositivity was an independent and significant prognostic factor of poor overall survival in their study (27). Kyzas et al. showed similar results in their study on head and neck squamous cell carcinoma (n=108). High dysadherin level was a significant independent prognostic factor for overall survival and correlated with higher clinical stage, lymph node metastasis, and increased intratumoral lymphatic invasion and density. Intense dysadherin immunostaining was mainly observed in the membranes of cancer cells but also in the basal cells of normal stratified squamous epithelium. Dysadherin-positive cancer cells were located especially in the areas with increased lymphatic concentration, surrounding and invading small intratumoral lymphatics (28).

Sato et al. investigated three different types of thyroid carcinomas (51 papillary, 10 follicular, and 31 undifferentiated carcinomas) and found significant increase in dysadherin protein level in undifferentiated carcinoma compared to papillary and follicular carcinomas and no staining in normal thyroid follicular epithelial cells. Dysadherin expression correlated with tumor size and metastasis, and patients who dies to thyroid carcinoma had higher tumoral dysadherin levels (31).

### Lung cancers

We found two lung cancer studies reporting high tumoral dysadherin protein level as an independent predictor of poor overall (32) or disease-free (33) survival (**Table 1** and **Supplementary Table 1**). Tamura et al. examined dysadherin protein by immunohistochemistry in patients with non-small cell lung cancer (NSCLC) (n=131). The overall survival was significantly worse for patients with dysadherin-positive tumors compared to those with dysadherin-negative tumors (31). The results were consistent with Ono et al. who studied stage I NSCLC specimens (n=107) and identified high dysadherin expression as predictor of poor disease-free survival. High dysadherin level together with low E-cadherin level could also be used to detect patients with lower overall or progression-free survival. No significant association between dysadherin and cancer recurrence was observed (32).

### Cancers of skin and connective tissue

Three immunohistochemical studies examined the role of dysadherin in skin and connective tissue cancers and found high tumoral protein level to predict poor overall survival (34–36) (**Table 1** and**Supplementary Table 1**). Nishizawa et al. reported that increased dysadherin level is an independent and significant prognostic factor for overall survival in patients with cutaneous malignant melanoma (n=115). High dysadherin expression also correlated with tumor subtype, Clark level, tumor thickness, ulceration, TNM stage, lymph node metastasis indicating dysadherin as a protumorigenic factor in this cancer. Dysadherin expression was also detected in basal cells of normal epidermis (34).

Izumi et al. examined epithelioid sarcomas and malignant rhabdoid tumors (n=72+6, respectively) and detected dysadherin-positive staining more frequently in proximal-type epithelioid sarcoma cases than in distal-type epithelioid sarcoma cases. In malignant rhabdoid tumors dysadherin staining was not observed. Patients with dysadherin positive epithelioid sarcoma survived for a significantly shorter time compared to those with dysadherin negative tumors (35). In another study by Izumi et al., dysadherin level was examined in synovial sarcomas (n=92) and similarly, patients with high dysadherin level survived for a significantly shorter time than those without dysadherin level. High dysadherin level correlated with age of the patient, glandularity and size of the tumor as well as Ki67-labeling index. Tumors with positive immunohistochemical staining also showed higher mRNA expression of dysadherin (36).

### Meta-analyses

**Figure 2:**
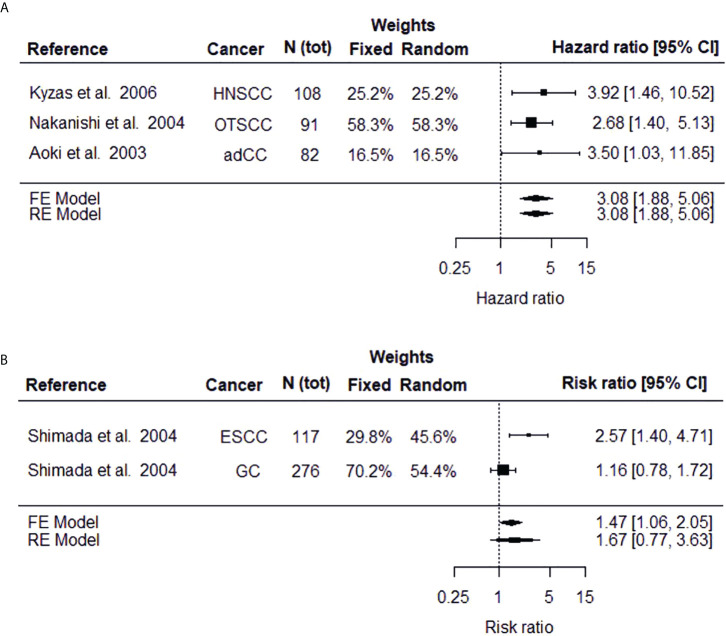
Forest plots depicting the meta-analysis results on high FXYD5 protein expression on risk of death estimated as hazard ratio **(A)** or risk ratio **(B)**. The black square displays the HR or RR identified in the study and the whiskers display the 95% confidence interval (CI). Size of the black box signifies the weight that the study contributes in the meta-analysis. The combined effect, estimated with both fixed and random effects models, is displayed as the black diamond.

The significance of dysadherin protein level (50% cut-off point) in estimating cancer patient’s overall survival was evaluated by two individual meta-analyses by combining the results of original studies which reported either HR (meta-analysis I) or RR (II) parameters. Meta-analysis I included results from three independent studies on head and neck (28) as well as oral tongue (27) squamous cell carcinoma and advanced colorectal carcinoma (14) (**Figure 2A**). The meta-analysis I revealed that cancer patients with high tumoral dysadherin protein level face a three times higher risk of death (HR 3.08, 95% CI 1.88-5.06, p-value <0.000, both fixed and random effects models) compared to patients with low dysadherin level. Heterogeneity between the included studies in this meta-analysis was low (Cochran Q = 0.447, p-value = 0.780, degrees of freedom = 2 and I2 = 0.00%) demonstrating the universal usability of this marker in evaluating cancer patient’s prognosis.

The relative risk (i.e., risk ratio) of death between patients with high (>50%) and low (<50%) tumoral dysadherin protein level have been studied in esophageal squamous cell carcinoma (22) and gastric cancer (15) and these studies were used for the meta-analysis II (**Figure 2B**). The results vary depending on the model used, especially on the significance (fixed effects model RR = 1.47, 95% CI 1.06-2.05, p-value = 0.023 and random effects model RR = 1.67, 95% CI 0.77-3.63, p-value = 0.197). For meta-analysis consisting of only few (typically 2 to 3) studies, the fixed effects model has been suggested as random effects estimators tend to overestimate the variance between studies (38, 39). To conclude the results of meta-analysis II, cancer patients with high dysadherin staining face a 1.5-times the risk of death compared to cancer patients with low tumoral dysadherin level, although the heterogeneity between studies is large (Cochran Q = 4.6365, p-value = 0.0313, degrees of freedom = 1 and I2 = 78.43%).

## Discussion

In this systematic review we gathered the available research articles on the prognostic role of dysadherin in cancer using PubMed, Scopus, Web of Science, clinical trial (clinicaltrials.gov, ICTRP) and pre-print (MedRXiv/BioRXiv and ResearchSquare) databases. The importance of biomarkers in the clinical management of cancer patients is increasing. A biomarker is any measurable biological molecule, such as DNA, RNA, protein or peptide, that can be used as an indicator of normal or abnormal biological state in an organism. Clinically, cancer biomarkers measure the risk of developing cancer, risk of cancer progression or response to therapy. Based on their usage, cancer biomarkers are classified into predictive and prognostic biomarkers. Predictive biomarkers provide information about the response to a particular therapy helping to identify the patients most likely to benefit from the treatment. Prognostic biomarkers are associated with the overall cancer outcome and indicate the likelihood of a future clinical event, such as death, disease recurrence or progression (40, 41). Reliability of a biomarker increases with the number of publications, yet as the cancer field is battling with the dramatic increase of information, a synthesis of the current knowledge is required. Systematic reviews, especially accompanied with meta-analyses, offer a solution for this need, but thorough approach is needed to ensure their good quality (42).

All 23 studies included in this systematic review found dysadherin to be a marker for poor prognosis either being directly associated with survival or markers indicating higher risk of malignancy. Furthermore, many studies (7/23) reported a connection between dysadherin and the occurrence of metastases. Dysadherin staining was mainly found on the plasma membrane of cancer cells. In some studies, the normal cells were found negative for dysadherin staining. However, in several cancer tissues (20, 24, 25, 27–29, 34–36) positive dysadherin staining was observed in the basal cells of normal epithelia, lymphocytes, and endothelial cells by using the same antibody (3) (see **Supplementary Table 1**). According to the studies published to date, the only cancer type negative for dysadherin staining was malignant rhabdoid tumor (35). Malignant rhabdoid tumor (RT) is a highly aggressive and lethal tumor that typically arise in brain, kidney or other soft-tissue type (43). In epithelioid sarcoma and malignant rhabdoid tumor, dysadherin is significantly useful for the differential diagnosis between these two tumor types (35).

Studies on cancer cell lines suggest that dysadherin overexpression results in reduced cell-cell adhesion and thereby increased metastasis potential (1, 8). Induction of cell motility by dysadherin may be both E-cadherin dependent and independent. Overexpression of dysadherin caused morphological changes *in vitro* and a dose-dependent down-regulation of E-cadherin by a posttranscriptional mechanism (3). On the other hand, dysadherin also seems to promote invasion and metastasis of cancer cells completely lacking E-cadherin (9). From the 23 articles included in this review, examining role of dysadherin in cancer prognosis, 13 also evaluated the level of E-cadherin by immunohistochemistry. In six studies, a substantial reverse association between increased dysadherin level and decreased E-cadherin level was observed. One study identified a tendency for this association (29) and one study showed diminished E-cadherin expression *in vitro* after silencing dysadherin (26). Likewise, increased dysadherin and reduced E-cadherin levels predicted the worst prognosis for the patients (18, 20, 27, 33, 36).

Limitations of the present study are the diversity of cancer types studied in the collected articles and relatively small number of total articles, which also affect the quality of meta-analysis (44) and, deter us from applying methods to analyze publication bias (45). Publication bias may still be present as all studies recognized dysadherin as prognostic factor for cancer, whereas negative results might have gone unpublished (46, 47). Furthermore, some relevant reports might have been missed since the snowball was not conducted. In addition, information on ethnicity was missing in the majority of studies, and the majority (~80%) of studies are conducted in Asian countries thus the results might not be applicable to other populations. Due to limited number of studies on the same cancer type now, meta-analyses could be only performed by combining studies of various cancers.

In conclusion, articles published to date consistently connect the high dysadherin levels or elevated mRNA expression with worse overall survival in various cancers. Mostly this phenomenon has been identified in colon and colorectal (n=3), ovarian (n=3) and head and neck cancer (n=3). For high dysadherin levels or mRNA expression to be a clinically relevant biomarker, clear parameters for high dysadherin protein level or mRNA expression should be uniformly defined. Furthermore, the studies, which look into the correlation between dysadherin and prognosis or patient parameters such as metastases, should focus on a certain cancer (sub)type. Using dysadherin protein level or mRNA expression to select cancer patients for different treatment paradigms is currently highly understudied topic in dysadherin research.

## Data availability statement

The original contributions presented in the study are included in the article/**Supplementary Material**. Further inquiries can be directed to the corresponding author.

## Author contributions

AN and KJ conceived and designed the work, performed the searches, analyzed the data and prepared the manuscript. MR assisted data analysis, commented and modified the manuscript and supervised the work. PÅ and TS contributed in supervising the work and modified the manuscript. All authors read and approved the manuscript and agree to be accountable for all aspects of the research in ensuring that the accuracy or integrity of any part of the work are appropriately investigated and resolved.

## Funding

This study received funding from the Medical Faculty of the University of Oulu and Oulu University Hospital special state support for research. PÅ received funding from the Academy of Finland (#308363).

## Acknowledgments

We thank the personnel of the library of University of Oulu for their help in conducting the literature search. We thank Dr. Hannu Vähänikkilä, Statistical services, Medical Faculty of the University of Oulu for his advice on meta-analysis.

## Conflict of interest

The authors declare that the research was conducted in the absence of any commercial or financial relationships that could be construed as a potential conflict of interest.

## Publisher’s note

All claims expressed in this article are solely those of the authors and do not necessarily represent those of their affiliated organizations, or those of the publisher, the editors and the reviewers. Any product that may be evaluated in this article, or claim that may be made by its manufacturer, is not guaranteed or endorsed by the publisher.

## Abbreviations

adCC, Advanced colorectal carcinoma;CSCC, Cervical squamous cell carcinoma; EC, Endometrial cancer; ECC, Extrahepatic cholangiocarcinoma; ESCC, Esophageal squamous cell carcinoma; HNSCC, Head and neck squamous cell carcinoma; HR, Hazard ratio; NSCLC, Non-small cell lung cancer; OC, Ovarian cancer; OTSCC, Oral tongue squamous cell carcinoma; PDAC, Pancreatic ductal adenocarcinoma; RR, Risk ratio or relative risk; SOC, Serous ovarian cancerons.
